# A highly efficient flexible dye-sensitized solar cell based on nickel sulfide/platinum/titanium counter electrode

**DOI:** 10.1186/1556-276X-10-1

**Published:** 2015-01-10

**Authors:** Gentian Yue, Xingping Ma, Weifeng Zhang, Fumin Li, Jihuai Wu, Guoqiang Li

**Affiliations:** Key Laboratory of Photovoltaic Materials of Henan, School of Physics and Electronics, Henan University, Kaifeng, 475004 China; Engineering Research Center of Environment-Friendly Functional Materials, Ministry of Education, Institute of Materials Physical Chemistry, Huaqiao University, Quanzhou, 362021 China

**Keywords:** Nickel sulfide, Platinum, Counter electrode, Flexible, Dye-sensitized solar cell

## Abstract

A composite film of nickel sulfide/platinum/titanium foil (NiS/Pt/Ti) with low cost and high electrocatalytic activity was synthesized by the use of an *in situ* electropolymerization route and proposed as a counter electrode (CE) catalyst for flexible dye-sensitized solar cells (FDSSCs). The FDSSC with the NiS/Pt/Ti CE exhibited a comparable power conversion efficiency of 7.20% to the FDSSC with the platinum/titanium (Pt/Ti) CE showing 6.07%. The surface morphology of the NiS/Pt/Ti CE with one-dimensional (1D) structure is characterized by using the scanning electron microscopy (SEM). The NiS/Pt/Ti CE also displayed multiple electrochemical functions of excellent conductivity, great electrocatalytic ability for iodine/triiodine, and low charge transfer resistance of 2.61 ± 0.02 Ω cm^2^, which were characterized by using the cyclic voltammetry (CV), electrochemical impedance spectroscopy (EIS), and Tafel polarization plots. The photocurrent-photovoltage (*J-V*) character curves were further used to calculate the theoretical optical light performance parameters of the FDSSCs. It may be said that the NiS/Pt/Ti counter electrode is a promising catalytic material to replace the expensive platinum in FDSSCs.

## Background

For the purpose of finding sustainable and renewable power, a lot of clean energy resources are developed recently; for instance, solar energy [1, 2], hydrogen energy [3, 4], and geothermal energy [5, 6] have been largely researched in order to substitute for the fossil fuel. Among them, the solar cell, an optical-to-electricity power conversion device, is one of the most important clean energy substitutions. Dye-sensitized solar cells (DSSCs) have been considered as the much potential next-generation photovoltaic devices for they are herein developed and exhibited advantages of feasibility, low cost, high efficiency, and so on [7–9]. Additionally, the flexible DSSC (FDSSC) has been drawn considerable attention and has been widely investigated recently, because its shape or surface can be devised and constructed, the technique of large-scale roll-to-roll processing, and rapid coating [10].

A FDSSC generally consists of a TiO_2_ film with a dye adsorbed as the working electrode, an electrolyte with the redox couple of iodide/triiodide (I^-^/I_3_^-^), and a catalyst counter electrode (CE). The CE, as the most important component in FDSSC, collects the electrons from the external circuit and catalyzes the reduction of I_3_^-^ to I^-^ between the CE and electrolyte interface. Thus, the materials with high conductivity for the electrons transporting, large specific surface area for the CE/electrolyte contact, and high catalytic activity for the reaction of I_3_^-^ to I^-^ are supposed to be preferred characteristics for the ideal CEs in FDSSCs. However, platinum (Pt) as an expensive noble metal provides an excellent performance, but the commercialization of DSSCs is impeded owing to high cost of Pt. Thus, fabrication of CEs with other cheaper materials is expected to bring down the production cost of the solar cells, especially in large-scale production. In this respect, the nickel sulfide with one-dimensional (1D) nanostructure and good electrocatalytic ability for the I^-^/I_3_^-^ redox couples was served as CEs in FDSSCs [11, 12]. Sun et al. [13] reported that NiS CE electrodeposited using a potential reversal technique showed high catalytic activity for the reduction of I_3_^-^ to I^-^. Ku et al. [14] prepared a highly transparent NiS CE by using an electrodeposition technique and presented a good photovoltaic performance for thiolate/disulfide mediated DSSCs. Therefore, NiS is a good candidate of an efficient CE for electronics, optoelectronics, and memory devices. Furthermore, it is well known that titanium foil (Ti) has been utilized to manufacture FDSSCs as anode and counter electrode materials for its flexibility, low sheet resistance, and superior corrosion resistances for I^-^/I_3_^-^ electrolyte [15, 16].

Herein, it is envisaged that a NiS/Ti counter electrode decorated with Pt nanoparticles by using a two-step cyclic voltammetry approach was used in FDSSCs for the first time. The NiS/Pt/Ti CE showed excellent electrocatalytic activity, stability, and low charge transfer resistance. The FDSSC fabricated with the NiS/Pt/Ti CE exhibited greatly improved performance of 7.20% under irradiation of 100 mW cm^-2^ (AM 1.5). The strategy of fabricating the NiS/Pt/Ti CE is rather simple, which can benefit the roll-to-roll production of FDSSCs on Ti foils.

## Methods

### Materials

The nickel (II) chloride hexahydrate (NiCl_2_ · 6H_2_O, 98%), thiourea (TU; ≥99.0%), acetone, acetonitrile, ethanol, polyethylene glycol with average molecular weight of 20,000 (PEG 20,000), 4-tert-butylpyridine (TBP), chloroplatinic acid, lithium perchlorate, Ti foils (0.25-mm thickness), and titanium tetrachloride (TiCl_4_) are purchased from Shanghai Chemical Agent Ltd., Shanghai, China. All reagents are of analytical reagent grade.

Conductive flexible plate (ITO/PEN flexible, sheet resistance 6 Ω cm^-2^, thickness 0.175 ± 0.05 mm, purchased from Shenzhen Huanan Xiangcheng Technology Co., Ltd., Shenzhen, China) was used as a substrate for precipitating TiO_2_ porous film and cut into 1 × 2 cm^2^ sheets carefully and ultrasonically cleaned sequentially in detergent, acetone, distilled water, and ethanol for 30 min, respectively, and then stored in isopropyl alcohol; the organometallic compound sensitized dye Z-907 *cis*-bis(isothiocyanato) (2,2′-bipyridyl-4,4′-dicarboxylato) (2,2′-bipyridyl-4,4′-di-nonyl) ruthenium (II) is obtained from Solaronix SA (Aubonne, Switzerland). Ti foils are used as the substrate due to their excellent conductivity, plasticity, and resistance to the corrosive electrolyte. Ti foils are cut into 1 × 2 cm^2^ carefully and ultrasonically cleaned sequentially in detergent, acetone, and distilled water for 30 min, respectively, then immersed in 0.2 mM hydrofluoric acid solution for 2 min and rinsed in distilled water again, and then stored in ethanol.

### Preparation of the NiS/Pt/Ti CE

The preparation of the NiS/Pt/Ti CE using a two-step cyclic voltammetry approach is outlined below. Firstly, the electrodeposition of NiS onto the Ti substrate was carried out with an electrochemical analyzer system (CHI660B, Shanghai Chenhua Device Company, Shanghai, China). All experiments were implemented in a three-electrode cell at room temperature (about 25°C), including one Pt foil as the counter electrode, one Ag/AgCl electrode as the reference electrode, and Ti substrate with an exposed area of 0.8 × 0.8 cm^2^ as the working electrode. The base polymerization solution consisted of 0.05 M NiCl_2_ and 1.0 M TU solution. A constant potential of -1.2 V vs. Ag/AgCl was employed for the electrodeposition of NiS on the Ti substrate. Similarly, the obtained NiS/Ti CE as the working electrode was soaked in 0.01 M H_2_PtCl_6_ and LiClO_4_ ethanol solution to carry out the electrodeposition procedure. The Ti foil substrate coated with NiS/Pt was put into anhydrous ethanol for 2 h and vacuum oven at 100°C for 12 h, respectively. For comparison, the Pt and NiS CEs were also prepared using a similar three-electrode system.

### Fabrication of FDSSC

**Figure 1:**
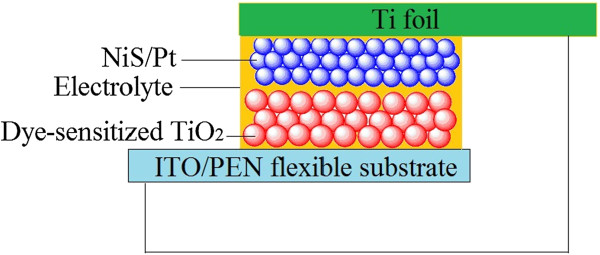
**Schematic of the Ti substrate flexible dye-sensitized solar cell.**

A TiO_2_ anode was prepared as described previously [17–19]. Briefly, a certain amount of treated P25, distilled water, and absolute ethanol were mixed with a mole ratio of 1:1:5, sonicated for 30 min before transferring to a 150-ml Teflon-lined autoclave (packing volume <80%), and then heated in an oven at 200°C for 24 h with no intentional control of ramping or cooling rate; thus, a homogeneous and stable TiO_2_ colloid was obtained. The TiO_2_ colloid with a particle size of 10 to 20 nm was coated on a ITO/PEN substrate by using doctor scraping technique. The resultant TiO_2_/ITO/PEN film was heated at 60°C in vacuum oven for 1 h. The process was repeated for two times to form a TiO_2_ film with the thickness of about 8 to 10 μm. Finally, the TiO_2_/ITO/PEN film was immersed in 0.3 mM of dye Z907 tert-butanol/acetonitrile solution for 24 h to absorb the dye adequately. Thus, a flexible dye-sensitized photoanode was obtained. The FDSSC was fabricated by injecting the liquid electrolyte (0.05 M of I_2_, 0.1 M of LiI, 0.6 M of tetrabutylammonium iodide, and 0.5 M of TBP in acetonitrile) in the aperture between the dye-sensitized TiO_2_/ITO/PEN electrode and the NiS/Pt/Ti CE (shown in Figure 1). The two electrodes were clipped together and wrapped with thermoplastic hot-melt Surlyn (Solaronix SA).

### Characterization

The surface morphology of the sample was observed by using JSM-7600 F field emission scanning electron microscope (SEM; JEOL Ltd., Akishima, Tokyo, Japan) with an energy-dispersive spectrometer (EDS) to obtain the information of the microstructures and the chemical compositions. Cyclic voltammetry (CV) and electrochemical impedance spectroscopy (EIS) were conducted by using a computer-controlled electrochemical analyzer (CHI 660B, CH Instrument, Austin, TX, USA). The electrolyte used in the photovoltaic test of FDSSC was also injected into the symmetric dummy cells for EIS measurements. EIS was carried out under the simulating open-circuit conditions at ambient atmosphere, sealing with thermoplastic hot-melt Surlyn and leaving an exposed area of 0.64 cm^2^. The frequency of the applied sinusoidal AC voltage signal was varied from 0.1 to 10^5^ Hz, and the corresponding amplitude was kept at 5 mV in all cases.

The photovoltaic test of FDSSC with an exposed area of 0.4 × 0.7 cm^2^ was carried out by measuring photocurrent-photovoltage (*J-V*) character curves under white light irradiation of 100 mW cm^-2^ (AM 1.5 G) from the solar simulator (XQ-500 W, Shanghai Photoelectricity Device Company, Shanghai, China) in ambient atmosphere. The fill factor (FF) and the photo-electric conversion efficiency (*η*) of FDSSC were calculated according to the following equations:
12

where *J*_sc_ is the short-circuit current density (mA cm^-2^), *V*_oc_ is the open-circuit voltage (V), *P*_in_ is the incident light power (mW cm^-2^), and *J*_max_ (mA cm^-2^) and *V*_max_ (V) are the current density and voltage at the point of maximum power output in the *J*-*V* curves, respectively.

The diffusion coefficient (*D*_*n*_) of CE in electrolyte was estimated in the light of the Randles-Sevcik equation as illustrated in Equation 3:
3

where *I*_pc_ is the cathodic current density, *K* is the constant of 2.69 × 10^5^, *n* means the number of electrodes contributing the charge transfer (here, *n =* 2), *A* is the area of the CE, and *C* and *v* represent the concentration of I_3_^-^ species and the scan rate, respectively.

The exchange current density (*J*_0_) and the limiting diffusion current density (*J*_lim_) for the Tafel curves could be estimated according to Equations 4 and 5:
45

where *R* is the gas constant, *n* (*n* = 2) is the number of electrons involved in the reduction of triiodide at the electrode, *D* is the diffusion coefficient of the triiodide, *l* is the spacer thickness, *T* is the temperature, *F* is Faraday's constant, *R*_ct_ is the charge transfer resistance, and *C* is the triiodide concentration.

## Results and discussion

### Surface morphology and composition of the samples

**Figure 2:**
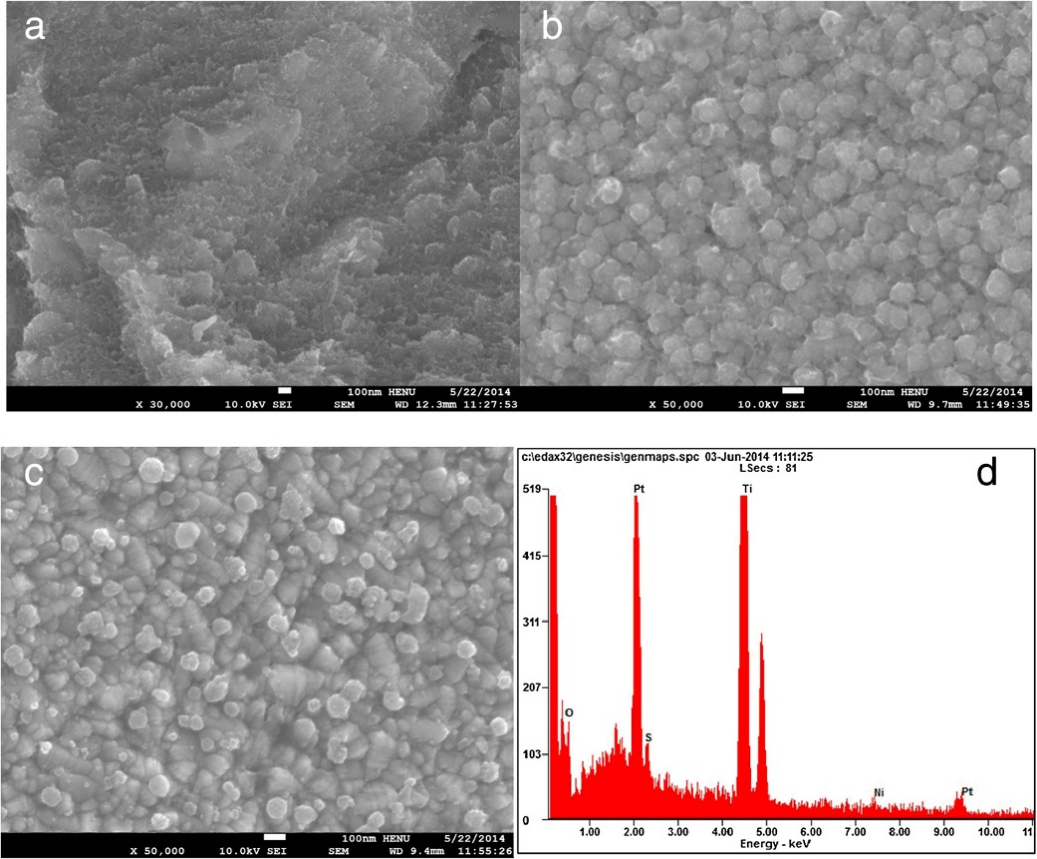
**SEM images of Ti foil (a), NiS/Ti (b), and NiS/Pt/Ti CEs (c) and EDS spectra of the NiS/Pt/Ti CE (d).**

Figure 2 represents the morphologies of the Ti foil, NiS/Ti, and NiS/Pt/Ti CEs and the EDS spectra of the NiS/Pt/Ti CE. Ti foil treated with HF solution exhibits a pleated sheet structure surface morphology as shown in Figure 2a, which provides a large surface area for the active materials to coat on. Figure 2b shows the SEM image of NiS/Ti CE, where the NiS nanoparticles uniformly arrange on the Ti substrate with perfect smooth surface. Figure 2c shows the top-view SEM image of the NiS/Pt/Ti CE. It is observed that the Pt nanoparticles are homogeneously dispersed with each other and deposited uniformly on the NiS/Ti surface. The NiS/Pt/Ti CE with a bump-like surface provides a highly effective contact area between the CE and the I^*-*^/I_3_^*-*^ electrolyte, thus possibly improving the penetration of I^-^/I_3_^-^ liquid electrolyte into the inside of the NiS/Pt/Ti film and ultimately creating highly electrocatalytic activity, and logically produces an enhanced performance for FDSSC.

The EDS analyses are carried out to further identify the compositions of the NiS/Pt/Ti CE. Figure 2d displays the presence of C, O, Ni, S, and Pt elements in the NiS/Pt/Ti film, in which the atomic percentages of Ni/S ratio is nearly 1:1. This illustrates that NiS was successfully deposited on Ti foil. Among Ti, O, Pt, and C elements, the large amount of Ti and Pt is responsible for the Ti foil substrate and electrodeposition of chloroplatinic acid, respectively; a little O may come from the passive oxide of TiO_2_ on the Ti foil substrate, and C is due to the holey conductive carbon glue. The results demonstrate that the NiS/Pt/Ti composite CE was successfully prepared by means of the facile electrodepositing route.

### Electrochemical properties

**Figure 3:**
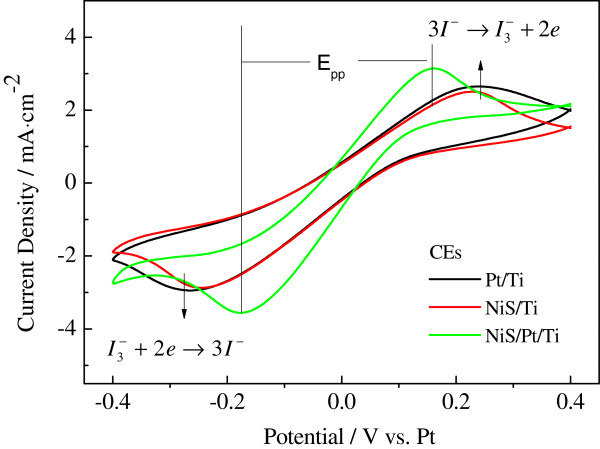
**Cyclic voltammograms for the Pt/Ti, NiS/Ti, and NiS/Pt/Ti counter electrodes with a scan rate of 50 mV s** ^**-1**^ **.**

To investigate the electrocatalytic ability of the various CEs, CV was carried out using a three-electrode electrochemical system. Figure 3 presents the CVs of the Pt/Ti, NiS/Ti, and NiS/Pt/Ti CEs under I^-^/I_3_^-^ electrolyte system in the potential interval of -0.4 to 0.4 V vs. Pt taken at a scan rate of 50 mV s^-1^ [20]. In the CV curves, the right set of anodic peaks correspond to 3I_2_ + 2*e*^-^ → 2I_3_^-^, and the left set of cathodic peaks correspond to I_3_^-^ + 2*e*^-^ → 3I^-^ [21], the cathodic peak current density (*I*_pc_) corresponds to the reaction rate of the catalyst for the reduction of I_3_^-^ ions to I^-^ ions. In general, a higher *I*_pc_ absolute value implies better electrocatalytic ability for the catalytic material; the peak-to-peak separation (*E*_pp_) is inversely correlated with the electrochemical rate constant of a redox reaction [22]. As shown in Figure 3 and Table 1, it is notable that the NiS/Pt/Ti CE displays much higher *I*_pc_ absolute value and more positive cathodic peak potential than the Pt/Ti and NiS/Ti CEs. These indicate that the electrocatalytic activity and conductivity of the NiS/Pt/Ti CE can be further enhanced for their excellent intrinsic electrocatalytic activity, electrical conductivity, and the synergistic catalytic effect of NiS and Pt [23]. These results are consistent with the performances of its corresponding cell.

**Table 1:** **The electrochemical performance parameters obtained from the EIS and CV based on various counter electrodes**

CEs	***R***_s_ (Ω cm ^2^)	***R***_ct_ (Ω cm ^2^)	***Z***_w_ (Ω cm ^2^)	|***I***_pc_| (mA cm ^-2^)	|***E***_pp_| (V)
Pt/Ti	5.02 ± 0.02	3.01 ± 0.02	0.62 ± 0.02	2.96 ± 0.01	0.50 ± 0.01
NiS/Ti	5.17 ± 0.02	3.10 ± 0.02	1.08 ± 0.02	2.88 ± 0.01	0.46 ± 0.01
NiS/Pt/Ti	4.87 ± 0.02	2.61 ± 0.02	0.33 ± 0.02	3.54 ± 0.01	0.33 ± 0.01

**Figure 4:**
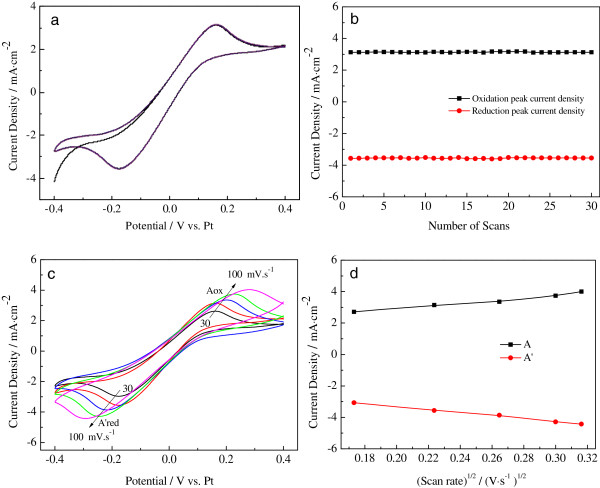
**Cyclic voltammograms.** Cyclic voltammograms of the NiS/Pt/Ti counter electrode with different scan rates **(a)** and the redox peak current versus square root of scan rates **(b)**, 30-cycle CVs of the NiS/Pt/Ti counter electrode with the scan rate of 50 mV s^-1^ **(c)**, and the relationship between the cycles and the maximum redox peak currents for the NiS/Pt/Ti electrode at the scan rate of 50 mV s^-1^ **(d)**.

Figure 4a,b presents 30-cycle continuous CVs of the NiS/Pt/Ti counter electrode and the relationship between the cycles and the maximum redox peak current densities for the NiS/Pt/Ti electrode at the rate of 50 mV s^-1^. In Figure 4a, the CVs of the NiS/Pt/Ti CE do not change but exhibit stable anodic and cathodic peak current densities with the consecutive 30-cycle test, and a good linear relationship between the cycle times and the maximum redox peak current densities is also presented in Figure 4b. This suggests that the NiS/Pt/Ti counter electrode there has an excellent electrochemical stability [24]. Figure 4c shows the effect of the scan rates on the CV property of the NiS/Pt/Ti CE in I^-^/I_3_^-^ electrolyte. With the increase of scan rate, the cathodic peak current densities and potentials gradually and regularly shift negatively, and the corresponding anodic peak current densities and potentials shift positively with increasing scan rates. A good linear relationship between cathodic and anodic peak current densities and the square root of the scan rates for the NiS/Pt/Ti CE is illustrated in Figure 4d. The result indicates that the adsorption of iodide species has little influence on the NiS/Pt/Ti CEs' surface, thus demonstrating no species interaction between the I^-^/I_3_^-^ redox couple and the NiS/Pt/Ti CE as well as the Pt CE [25, 26]. Furthermore, according to Equation 3 [27], the reduction reaction of I^-^/I_3_^-^ that occurred at the Pt/Ti, NiS/Ti, and NiS/Pt/Ti CEs belongs to the diffusion-controlled transport process, and *D*_*n*_ can be estimated. Thus, the diffusion coefficients of I_3_^-^ for the Pt/Ti, NiS/Ti, and NiS/Pt/Ti CEs are estimated to be 3.16 × 10^-6^, 3.01 × 10^-6^ and 5.53 × 10^-6^ cm^-2^ s^-1^, respectively. The diffusivity for the NiS/Pt/Ti CE is much larger than that of the Pt/Ti and NiS/Ti CEs, probably originating from its internal properties and the improvement of the surface roughness of the NiS/Pt/Ti CE.

**Figure 5:**
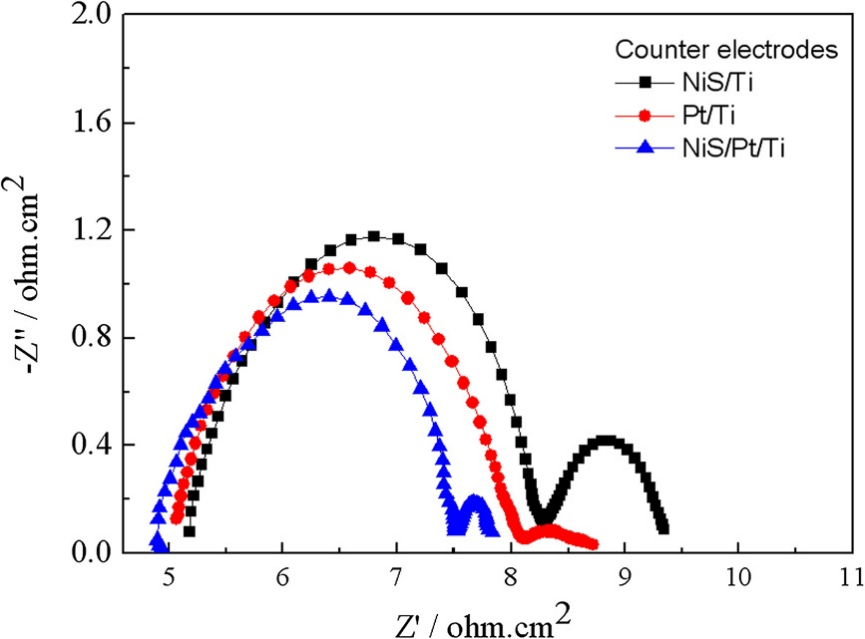
**EIS of the symmetrical Pt/Ti, NiS/Ti, and NiS/Pt/Ti CEs for I** ^**-**^ **/I**_**3**_^**-**^ **redox couple.**

EIS measurements were carried out, using a symmetric dummy cell to further get the deep insights into the electrocatalytic activities of the CEs for I^-^/I_3_^-^ electrolyte. Figure 5 shows the Nyquist plots of the various symmetrical Pt/Ti, NiS/Ti, and NiS/Pt/Ti CEs. The high-frequency intercept on the real axis represents the series resistance (*R*_s_) including the sheet resistance of two identical CEs and the electrolytic resistance; the first semicircle at high frequency refers to the charge transfer resistance (*R*_ct_) for the I_3_^-^ reduction at the CE|electrolyte interface, and the second semicircle at low frequency represents the Nernst diffusion impedance (*Z*_*w*_) corresponding to the diffusion resistance of the I^-^/I_3_^-^ redox species, respectively [28, 29]. The EIS parameters obtained from the Nyquist plots are listed in Table 1. The *R*_s_ value is a positive correlation with the adhesion of the electrocatalytic materials and the Ti foil (discussed at the anterior CV section). The *R*_s_ of Pt/Ti, NiS/Ti, and NiS/Pt/Ti CEs are found to be 5.02 ± 0.02, 5.17 ± 0.02, and 4.87 ± 0.02 Ω cm^2^, respectively, indicating a firm adhesion on the Ti substrate for the abovementioned catalysts. The *R*_ct_ of NiS/Pt/Ti CE is 2.61 ± 0.02 Ω cm^2^ much lower than that of Pt/Ti (3.01 ± 0.02 Ω cm^2^) and NiS/Ti (3.10 ± 0.02 Ω cm^2^) counter electrodes for a synergistic effect of the Pt and NiS nanoparticles [30]. The *Z*_*w*_ of NiS/Pt/Ti CE (0.33 ± 0.02 Ω cm^2^) is smaller than that of Pt/Ti (0.62 ± 0.02 Ω cm^2^) and NiS/Ti (1.08 ± 0.02 Ω cm^2^) CEs. The *Z*_*w*_ is remarkably reduced in the I^-^/I_3_^-^ redox electrolyte when the Pt nanoparticles are combined with NiS, indicating the improvement of electrolyte penetration in CEs [31]. Briefly, the Pt nanoparticles with excellent catalytic activity and high electrical conductivity not only provide the activity surface area for I^-^/I_3_^-^ redox couple but also improve the electron transport. The enhanced electrocatalytic activity can make electrons transmit across the CE|FTO interface easily, which hopefully provides a considerable enhancement on the photovoltaic performance.

**Figure 6:**
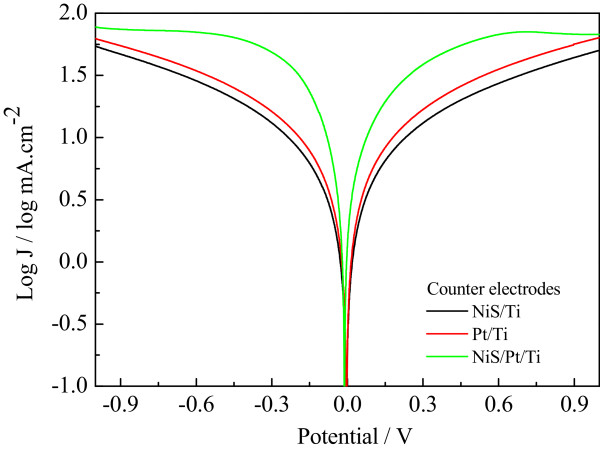
**Tafel curves of the symmetrical Pt/Ti, NiS/Ti, and NiS/Pt/Ti counter electrodes.**

Figure 6 shows the Tafel curves of the Pt/Ti, NiS/Ti, and NiS/Pt/Ti CEs in I^-^/I_3_^-^ electrolyte to further aid the investigation of the interfacial charge-transfer properties of the I^-^/I_3_^-^ redox couple on the CEs. They show the logarithmic current density (log*J*) as a function of the potential for the reduction of I^-^/I_3_^-^. In theory, the curve at low potential curve (|U| < 0.120 V) corresponds to the polarization zone, the region at middle potentials (with steep slopes) represents the Tafel zone, and the region at high potentials (horizontal portion) represents the diffusion zone [32]. The exchange current density (*J*_0_) is obtained as the intercept of the extrapolated linear region of the curve when the over-potential is zero; *J*_0_ is tantamount to the catalytic activity of the electrode. The limiting diffusion current density (*J*_lim_) can be obtained in the curve at high potential (horizontal part). Thus, according to the slopes for the anodic or cathodic branches, *J*_0_ follows the order of NiS/Pt/Ti > Pt/Ti > NiS/Ti, implying an excellent electrocatalytic activity for the NiS/Pt/Ti CE in I^-^/I_3_^-^ redox couple. The relationship between *J*_0_ and *R*_ct_ of an electrode for the reduction of I_3_^-^ ions to I^-^ ions can also be calculated by Equation 4. Moreover, the NiS/Pt/Ti CE also provides an excellent *J*_lim_ and a large diffusion coefficient in the diffusion zone, which is determined by the diffusion of the I^-^/I_3_^-^ redox couple in electrolyte [32]. Based on Equation 5, a smaller diffusion coefficient (*D*) value for NiS/Pt/Ti CE is calculated among the abovementioned CEs, and this proves that the NiS/Pt/Ti CE holds a fast diffusion velocity of the redox couple in the electrolyte. Thus, the results for the electrocatalytic activity derived from the Tafel polarization and EIS data are well consistent and can be logically expected to considerably improve the photovoltaic performance for FDSSC [33].

### Photovoltaic performance of FDSSCs with NiS/Pt/Ti CE

**Figure 7:**
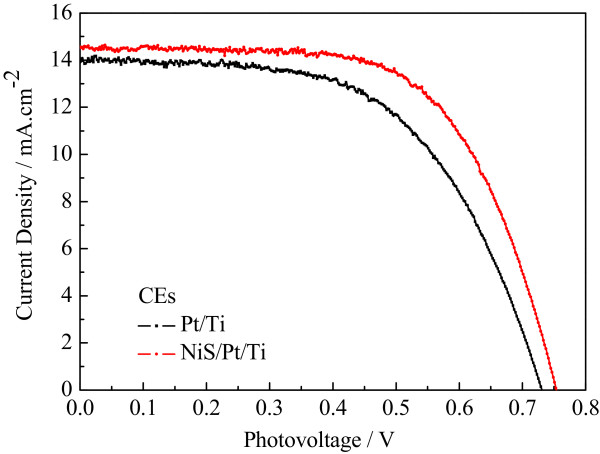
***J-V*** **characteristics of the FDSSCs fabricated with different CEs under the standard illumination.**

Figure 7 shows the photocurrent density-voltage (*J-V*) curves for FDSSCs based on the Pt/Ti and NiS/Pt/Ti CEs under the irradiation of 100 mW cm^-2^, and the corresponding photovoltaic parameters are presented in Table 2. The FDSSC with the Pt/Ti CE obtained *J*_sc_ of 13.86 mA cm^-2^, *V*_oc_ of 0.73 V, FF of 0.60, and corresponding to the *η* of 6.07%. The photovoltaic performance parameters of the FDSSC based on the NiS/Pt/Ti CE are *J*_sc_ of 15.55 mA cm^-2^, *V*_oc_ of 0.75 V, FF of 0.66, and *η* of 7.20%, respectively. The improved performance of the FDSSC with NiS/Pt/Ti CE is essentially attributed to the large contact area at the interface of the CE and electrolyte, the highly electrocatalytic ability and superior conductivity provided by the synergistic catalytic effect of the Pt and NiS nanoparticles, and the enhanced diffusion velocity for I^-^/I_3_^-^ redox couple as indicated in the aforementioned SEM, CV, EIS, and Tafel polarization plots.

**Table 2:** **The photoelectric property parameters of the FDSSCs with Pt/Ti and NiS/Pt/Ti counter electrodes**

FDSSCs	***V***_oc_ (V)	***J***_sc_ (mA cm ^-2^)	FF	***η***(%)
Pt/Ti-based	0.73	13.86	0.60	6.07
NiS/Pt/Ti-based	0.75	14.55	0.66	7.20

## Conclusions

A NiS/Ti counter electrode decorated with Pt nanoparticles was successfully synthesized by using a facilitated electrodeposition approach onto the Ti substrate and served as the counter electrode catalyst for flexible dye-sensitized solar cells. Ti foil treated with HF solution exhibits a pleated sheet structure surface morphology which provides a large interface contact between the electrolyte and the NiS/Pt/Ti catalyst. Thus, the NiS/Pt/Ti CE with 1D nanostructure displays multiple functions, i.e., low cost, large surface area, efficiency, excellent conductivity, and great electrocatalytic ability for iodine/triiodine and lower charge transfer resistance of 2.61 ± 0.02 Ω cm^2^ compared to the Pt/Ti electrode (3.01 ± 0.02 Ω cm^2^). The CV curves of the Pt/Ti, NiS/Ti, and NiS/Pt/Ti CEs show that their electrocatalytic ability are in the sequence of NiS/Pt/Ti > Pt/Ti > NiS/Ti. Compared to the Pt/Ti and NiS/Ti CEs, the NiS/Pt/Ti CE obviously shows an improved electrocatalytic activity for the synergistic effects of the NiS with the fast electron transfer and Pt nanoparticles with highly electrocatalytic activity. Moreover, the Pt nanoparticles highly dispersed on NiS/Ti surface provides more active sites for I_3_^-^ reduction and facilitates the redox electrolyte diffusion within the CE. Therefore, the FDSSC based on the NiS/Pt/Ti CE exhibits much higher power conversion efficiency of 7.20% than that of the FDSSC with the Pt/Ti CE (6.07%) under the illumination of 100 mW cm^-2^. Consequently, the NiS/Pt/Ti CE prepared by using a simple electropolymerization technique indicates a great promising catalytic material as low-cost and high-performance alternative in large-scale FDSSCs.

## Abbreviations

CE: counter electrode
CV: cyclic voltammetry
*D*_*n*_: the diffusion coefficient
FDSSC: flexible dye-sensitized solar cell
I^-^/I_3_^-^: iodide/triiodide
*J*_0_: exchange current density
*J*_lim_: limiting current density
*J*_ma*x*_: maximum current density
*J*_sc_: short-circuit current density
*J-V*: photocurrent-photovoltage
NiS: nickel sulfide
*P*_in_: incident light power
PCE: power conversion efficiency
*R*_ct_: charge transfer resistance
*R*_s_: series resistance
SEM: scanning electron microscopy
Ti: titanium foil
TPB: 4-tert-butylpyridine
*V*_max_: maximum voltage
*V*_oc_: open-circuit voltage
*Z*_*w*_: finite layer Nemst diffusion impedance.
